# Medicinal herbs: Potential polypills in cardiovascular diseases

**DOI:** 10.1111/jch.14203

**Published:** 2021-02-26

**Authors:** Samaneh Noroozi, Arman Zargaran, Mehrdad Karimi

**Affiliations:** ^1^ Department of Traditional Medicine School of Persian Medicine Tehran University of Medical Sciences Tehran Iran; ^2^ Department of Traditional Pharmacy School of Persian Medicine Tehran University of Medical Sciences Tehran Iran

**Keywords:** cardiovascular, herb, polypill

## 1 MEDICINAL HERBS: POTENTIAL POLYPILLS IN CARDIOVASCULAR DISEASES

I have read the review article entitled “The feasibility of polypill for cardiovascular disease prevention in Asian Population” by Apichard Sukonthasarn and co‐authors published in *The Journal of Clinical Hypertension* (2020; on line version).^1^ I want to congratulate the authors for this successful review article and present idea.

The past decade, drug discovery has been moved from one drug‐one target level to computational multi‐target level. Based on this paradigm, a multi‐target drug could offer beneficial synergistic effects for complex diseases such as cardiovascular diseases. Medical herbs featuring multi‐constituents, multi‐targets, and multi‐effects are valuable resources for multi‐target drug discovery.^2^, ^3^, ^4^, ^5^ Approximately two‐thirds of the world's plant species are widely used in medicines.^5^, ^6^

The cardiovascular drugs such as aspirin, digitalis, and verapamil have been derived from plants.^5^, ^7^ Traditional systems of medicines have potential to contribute in future drug discovery. In traditional medicine, a single herbal medicine characterized as multi‐components and multi‐functions produces the desired pharmacological effects.^5^, ^8^

Persian medicine is one of the oldest medical schools in which medical herbs such as chamomile, lemon balm, and phyllanthus emblica containing biologically active constituents for cardiovascular diseases have been used. The beneficial effects of specific herbal extracts for cardiovascular disease depend on its complex constituents and interactions that can target several signaling pathways.^9^ For example, chamomile featured as a multi‐component acts through multiple mechanisms including anti‐inflammation, antioxidation, vasorelaxation, glycemic and lipid profile control, and anti‐platelet to synergistically benefit patients with ischemic heart disease, or at risk for it.^10^, ^11^, ^12^ We speculate that clinical knowledge of persian medicine could provide promising framework from specific medical herb for multi‐component, multi‐target drug, or polypill design.

## CONFLICT OF INTEREST

There are no conflicts of interest to disclose.

## AUTHOR CONTRIBUTIONS

Karimi, M contributed to design, critically revised the manuscript, gave final approval, and agreed to be accountable for all aspects of work ensuring integrity and accuracy. Noorozi, S contributed to conception and design, drafted the manuscript, critically revised the manuscript, gave final approval, and agreed to be accountable for all aspects of work ensuring integrity and accuracy. Zargaran, A contributed to conception, critically revised the manuscript, gave final approval, and agreed to be accountable for all aspects of work ensuring integrity and accuracy.
